# Ultrasound-Assisted Maillard Glycosylation of *Zophobas morio* Protein–Maltodextrin Conjugates: Effects on Structure and Acid-Induced Gel Properties

**DOI:** 10.3390/gels12050391

**Published:** 2026-05-02

**Authors:** Ha Seong Cho, St. Nur Hikmah, Niken Larasati Kusumawardani, Won Young Lee

**Affiliations:** 1 School of Food Science and Technology, Kyungpook National University, Daegu 41566, Republic of Korea; hasung31694@knu.ac.kr (H.S.C.); drstnurhikmah@gmail.com (S.N.H.); nikenlarasatik@gmail.com (N.L.K.); 2 Research Institute of Tailored Food Technology, Kyungpook National University, Daegu 41566, Republic of Korea; 3 Department of Biomedical Technology, School of Convergence, Kyungpook National University, Daegu 41566, Republic of Korea

**Keywords:** *Zophobas morio* protein, ultrasound-assisted, Maillard glycosylation, maltodextrin, acid-induced gel

## Abstract

In this study, we investigated the effect of ultrasound-assisted Maillard glycosylation reaction time on the structural, physicochemical, and acid-induced gel properties of *Zophobas morio* protein–maltodextrin (ZMP–MD) conjugates. Ultrasound treatment up to 45 min (100 kHz, 450 W, 70 °C) significantly accelerated the conjugation efficiency (15.81%) compared to that of wet heating at 70 °C for 6 h (13.62%) (*p* < 0.05). Fourier transform infrared spectroscopy (FT-IR) and sodium dodecyl sulfate-polyacrylamide gel electrophoresis (SDS-PAGE) analyses confirmed that both Maillard glycosylation methods formed ZMP–MD conjugates. In addition, the results for secondary structure, surface hydrophobicity, and zeta potential revealed that the ultrasound treatment promoted greater protein structural unfolding, decreasing α-helix while increasing β-sheet and random coil content compared to wet heating. These changes in structural and physicochemical properties of ZMP–MD conjugates impacted the glucono-δ-lactone (GDL)-based acid-induced gel properties. Even though Maillard glycosylation with MD weakened gel properties compared to native ZMP, the gel obtained after 45 min of ultrasound treatment exhibited a higher storage modulus, gel strength, and water-holding capacity than the wet-heated ZMP–MD gel. In conclusion, these findings suggest that properly controlled ultrasound-assisted Maillard glycosylation can modify protein structure, potentially improving its gel properties.

## 1. Introduction

Super mealworm (*Zophobas morio*) is one of the most promising edible insects as an alternative nutrient resource due to its nutritional profile (39–50% protein, 28–43% fat, and 0.7–8.17% ash), and its notable environmental and economic advantages [1,2]. This species belongs to the darkling beetle family, and its appearance is similar to that of the mealworm (*Tenebrio molitor*), but it is larger and longer. [3]. In terms of protein quality, *Zophobas morio* protein (ZMP) exhibited a higher essential amino acid index (EAAI) than traditional proteins, including fish meal and soy, and it is comparable to casein [4]. In addition, ZMP exhibited techno-functional properties similar to those of *Gryllus asimillis* and *Acheta domesticus*, including water- and oil-holding capacity and foaming and emulsifying properties [5]. Moreover, several studies have reported that significant protein digestibility, antioxidant, and anti-inflammatory activities of ZMP [6,7,8]. Based on these benefits, ZMP, as an alternative protein, has been applied to develop familiar products (e.g., muffins, batters, and sausages), demonstrating its applicability to insect-based foods [9,10,11]. Considering these advantages of ZMP, its use in food fortification and in the pharmaceutical field is expected to increase in the future.

Gelation is a significant property of edible insect proteins, which can enhance food sensory quality and texture and deliver bioactive compounds [12]. Among gelation methods, including heat-, acid-, salt-, and enzyme-induced, acid-induced gelation has been widely applied due to its simplicity, ease of application, and ability to load heat-sensitive compounds [13,14,15]. A common acid-induced gelation is conducted in the system containing glucono-δ-lactone (GDL). The addition of GDL to the protein solution reduces electrostatic repulsion, promoting aggregation and gelation near the isoelectric point. The gel network is primarily built through non-covalent interactions, with possible contributions from covalent bonds, such as disulfide linkages [15]. Thus, protein interactions are critical parameters for building a three-dimensional network and are highly related to gel properties.

Over the last decade, the Maillard glycosylation reaction has been studied as a non-enzymatic chemical reaction to improve the techno-functional properties of edible insect proteins [16,17,18]. The glycosylation approach induces the coupling of an amino group of the protein with the carbonyl group of a reducing sugar under heating conditions [19]. During glycosylation, reducing sugars are covalently attached to proteins, leading to structural unfolding and the exposure of hydrophobic residues, which promote rapid adsorption at the air–water interface and, consequently, enhance water solubility, emulsifying properties, and foaming capacity [20]. Furthermore, this method uses a naturally occurring reaction and does not require chemical reagents, making it a safe, simple, and effective way to improve the functional properties of proteins [21]. Maltodextrin (MD) is a hydrolysis product of starch, which has a dextrose equivalent (DE) level below 20 and is composed of α-(1, 4) and α-(1, 6) linked D-glucose polymers, oligomers, or both [22]. It shows significant flexibility and good dispersion in aqueous solution and has a neutral charge, preventing electrostatic complexation between the protein and sugar [23]. Owing to its unique properties, MD has been widely used for glycosylation with proteins [14,24].

The wet heating method is a conventional glycosylation method for producing protein-polysaccharide conjugates, which leads to the Maillard reaction (MR) in a liquid state under heating conditions [25]. However, this approach requires a long reaction time, from several hours to days, which affects glycosylation efficiency [26]. In addition, this method often results in excessive product browning and denatured protein aggregation at high temperatures, limiting its suitability for industrial protein modification [27]. In recent years, the application of ultrasound technology to glycosylation has attracted attention due to its ability to accelerate the MR [28]. The acoustic cavitation effect of ultrasound generates rapid bubble formation, growth, and collapse, producing intense mechanical forces, including shear forces, shock waves, and turbulence. These effects can enhance the contact between reactive groups of proteins and polysaccharides, thereby accelerating glycosylation and further inducing conformational changes in the protein-polysaccharide conjugates, ultimately leading to improved functional properties [29]. Zhao et al. [14] reported that the acid-induced gel properties of soy protein isolate can be enhanced by incorporating ultrasound treatment followed by Maillard glycosylation with MD. They found that appropriate ultrasound treatment increased the surface hydrophobicity, free sulfhydryl groups, and particle size of the conjugate, resulting in higher water-holding capacity, gel strength, and a denser microstructure than the solely heat-induced conjugate. However, no study has compared a direct ultrasound-assisted Maillard glycosylation of edible insect protein with MD to the conventional wet heating method. Moreover, the effects of these two approaches on the structural and acid-induced gel properties of insect proteins have not been compared.

Therefore, this study aimed to evaluate the efficiency of direct ultrasound-assisted glycosylation compared with conventional wet heating for the production of *Zophobas morio* protein–maltodextrin (ZMP–MD) conjugates. Furthermore, the effects of these two methods on the structural, physicochemical, and acid-induced gel properties of ZMP–MD conjugates were comparatively examined.

## 2. Results and Discussion

### 2.1. Effect on the Degree of Grafting (DG)

**Figure 1:**
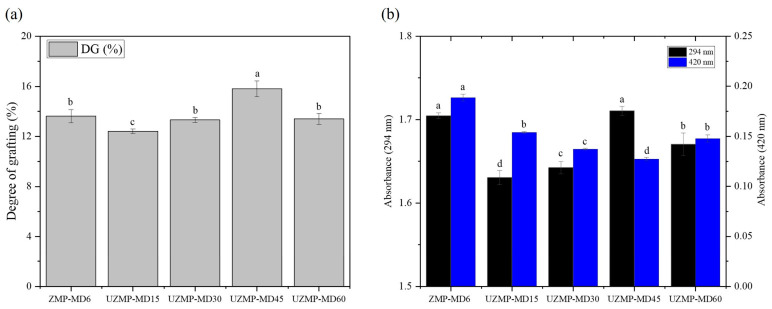
(**a**) The degree of grafting (DG) and (**b**) absorbance at 294 nm (glycosylation intermediates) and 420 nm (browning index) of the ZMP–MD6 conjugate and UZMP–MD15, UZMP–MD30, UZMP–MD45, and UZMP–MD60 conjugates. Different lowercase letters indicate significant differences (*p* < 0.05) among all samples for each parameter.

The DG of ZMP–MD conjugates produced by the conventional wet heating (6 h) and ultrasound-assisted Maillard glycosylation at different times (15, 30, 45, and 60 min) is presented in Figure 1a. As a result, ultrasound treatment at short times significantly accelerated the Maillard glycosylation between ZMP and MD. For example, the DG of UZMP-MD45 (15.81%) was higher than that of ZMP-MD6 (13.62%), suggesting enhanced grafting efficiency by ultrasound. The enhancement of the reaction could be attributed to acoustic cavitation induced by ultrasound [21]. Cavitation generates high shear stress, which gradually unfolds protein structures and exposes more active amino groups on the surface, thereby facilitating grafting between ZMP and MD [26]. At the same time, the rapid formation and collapse of cavitation bubbles in liquid systems also resulted in strong turbulence, leading to effective heat and mass transport and contributing to the formation of conjugates with MD [28]. However, UZMP–MD60 exhibited a significant decrease in DG (12.80%) compared to UZMP–MD60 (*p* < 0.05). This result indicates that prolonged ultrasound time can denature proteins, leading to protein aggregation and reduced grafting efficiency [30]. Consequently, an appropriate ultrasound-assisted Maillard glycosylation time efficiently promotes the covalent bonding between proteins and polysaccharides compared with wet heating.

### 2.2. Glycosylation Intermediates and Browning Index

**Figure 2:**
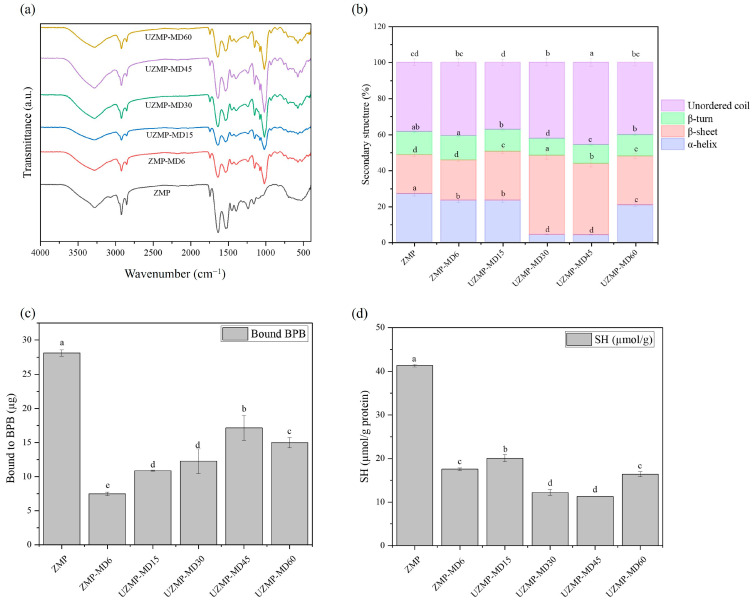
(**a**) The Fourier transform infrared spectroscopy, (**b**) secondary structure contents, (**c**) surface hydrophobicity, and (**d**) free sulfhydryl (R-SH) content of ZMP, ZMP-MD6 conjugate, and UZMP-MD15, UZMP-MD30, UZMP-MD45, and UZMP–MD60 conjugates. Different lowercase letters indicate significant differences (*p* < 0.05) among all samples for each parameter.

The Maillard glycosylation involves a condensation reaction between proteins and polysaccharides during heating. Thus, the absorbance at 294 nm (A_294_) and 420 nm (A_420_) correlates with intermediates (Amadori compounds) and the advanced-stage Maillard reaction products, respectively [31]. Figure 2b shows both the intensity of the glycosylation intermediates and the browning index of ZMP–MD conjugates. Notably, extending ultrasound treatment up to 45 min increased the A_294_ value to a level comparable to that of ZMP–MD6, followed by a slight decrease at 60 min. In contrast, the A_420_ value gradually decreased with increasing ultrasound time up to 45 min, but increased again at 60 min. These observations suggest that ultrasound treatment may condense glycosylation intermediates during the Maillard reaction but reduce the formation of brown products [32]. This may be due to the diverse ultrasound effects involved, including higher temperature, turbulence, reactive radicals, and shear stress [33]. Thus, cavitation effects may interfere with the formation of end-stage Maillard reaction products by inhibiting the polymerization of intermediates [34]. These findings align with a previous study by Zhao et al. [14], who reported that the browning intensity of soy protein–MD conjugates produced by ultrasound treatment was lower than that of the conjugates obtained by traditional wet heating. Additionally, prolonged ultrasound treatment may trigger protein aggregation by exposing hydrophobic residues, thereby reducing their interaction with MD [30]. This is in agreement with the observed absorbance values and grafting degree of UZMP–MD60. Therefore, appropriate ultrasound-assisted Maillard glycosylation conditions may enable the production of protein–polysaccharide conjugates with low browning intensity.

### 2.3. Structural Properties of ZMP–MD Conjugates

#### 2.3.1. Fourier Transform Infrared Spectroscopy (FT-IR)

FT-IR analysis was conducted to investigate the effects of the conventional wet heating and ultrasound-assisted Maillard glycosylation on the functional groups of ZMP–MD conjugates. The spectra of ZMP and all conjugates from 400 to 4000 cm^−1^ are shown in Figure 2a. The ZMP exhibited characteristic amide absorption bands, with an amide I band at 1634 cm^−1^ (C=O stretching) and peaks at 1533 cm^−1^ (amide II) and 1237 cm^−1^ (amide III) corresponding to N–H bending and C–N stretching vibrations [7]. After conjugation with MD, the intensities of those groups decreased in all conjugates, suggesting the loss of amino groups in the native ZMP during the Maillard reaction [35]. Notably, the newly adsorbed peaks at 1036 cm^−1^ in all conjugates were observed, which could be attributed to the stretching vibration of the C-O-C glycosidic bond [36]. In addition, at this wavenumber, the intensity of the UZMP–MDs increased with ultrasound time, which could be related to the grafting of functional groups from MD onto ZMP [28]. All conjugates showed some new group peaks around 760 cm^−1^, 931 cm^−1^, and 1079 cm^−1^ compared to the native ZMP. These peaks originated from Maillard reaction products, such as Schiff bases, Amadori compounds, and pyrazines, which have been reported in previous studies [36]. Overall, the wet heating and the ultrasound-assisted Maillard glycosylation had similar effects on the primary structure of ZMP–MD conjugates.

#### 2.3.2. Circular Dichroism (CD)

Circular dichroism is a sensitive method for identifying changes in the secondary structure of proteins. The secondary structure contents of ZMP, ZMP–MD, and UZMP–MDs are shown in Figure 2b and Table 1. For ZMP, the contents of α-helix, β-sheet, turn, and random coil were 26.97%, 21.67%, 12.80%, and 38.63%, respectively. After conjugation with MD, either wet heating or ultrasound-assisted treatment up to 45 min showed a decrease in the content of α-helix, whereas an increase in the content of β-sheet and random coil compared to the native ZMP. These changes could be attributed to covalent bonding between ε-amino groups in the α-helix region of ZMP, driving the transformation from α-helix to β-sheet and random coil structures [20]. Moreover, UZMP–MD45 exhibited a greater loss of α-helix (4.10%) but higher β-sheet (39.60%) and random coil (45.80%) contents compared to those of ZMP–MD6 (23.37%, 22.40%, and 40.70%). These differences were statistically significant (*p* < 0.05). Such changes were caused by the strong mechanical force exerted by cavitation bubbles, leading to the breaking of hydrogen bonding and partial unfolding of the helical structure [28]. In addition, these alterations in ZMP structure by ultrasound treatment promoted more favorable conditions for grafting MD onto ZMP, which could be related to the observed higher DG values for the UZMP–MD 45 (Figure 1a). However, UZMP–MD60 displayed an increase in α-helix and a decrease in β-sheet and random coil contents, suggesting that the thermal effect from the lasting ultrasound treatment may cause protein reaggregation rather than remaining in its unfolded state [37]. In summary, the analysis of CD confirms that ultrasound-assisted Maillard glycosylation can induce more structural transformations than the wet heating method and result in differences in the functionality of conjugates.

#### 2.3.3. Surface Hydrophobicity and Free Sulfhydryl (-SH) Content

Surface hydrophobicity and -SH groups on the protein surface are key factors in the formation of protein networks during acid-induced gelation. The effects of the wet heating and ultrasound-assisted Maillard glycosylation on hydrophobicity and free -SH groups of ZMP–MD conjugates are shown in Figure 2c,d. As shown in Figure 2c, the conjugation with MD seemed to inhibit the accessibility of BPB to the hydrophobic residues of ZMP. The hydrophobicity of ZMP–MD6 (7.46) was significantly lower compared to the original ZMP (28.12) (*p* < 0.05). This could be due to the insertion of numerous hydrophilic hydroxyl groups from MD into ZMP, thereby decreasing hydrophobicity [36]. Nevertheless, UZMP–MD15, 30, and 45 showed a significant increase in hydrophobicity, from 10.87 to 17.15 (*p* < 0.05), which could be explained by protein unfolding or dissociation of protein aggregates under ultrasound, thereby exposing buried hydrophobic groups [38]. The obtained results could further support the CD analysis, which showed that the ultrasound treatment induced protein unfolding (Table 1). In addition, these trends are consistent with a previous study by Ma et al. [20], which reported that ultrasound-assisted Maillard glycosylation enhanced surface hydrophobicity, together with the extension of the protein molecular structure. However, UZMP–MD60 promoted a significant (*p* < 0.05) re-decrease in surface hydrophobicity (14.99) compared to UZMP–MD45, which may be due to the high-temperature environment caused by excessive treatment. Thus, the molecule may aggregate, leading to reconstitution and the formation of an internal hydrophobic region [39].

The content of free -SH groups in all conjugates obtained from either wet heating or ultrasound-assisted Maillard glycosylation was significantly lower than that in the native ZMP (*p* < 0.05) (Figure 2d). Reduction in the amount of the free -SH in ZMP–MD6 may be because the free -SH groups on the surface of ZMP tend to react with Schiff bases and Amadori rearrangement products from Maillard glycosylation [19]. In addition, a more gradual reduction in the free -SH content in UZMP–MD30 and 45 was observed. The turbulent flow and shear forces from ultrasound induce unfolding of proteins, exposing buried -SH groups to the surface, which readily react with free radicals generated by cavitation, leading to oxidation of the exposed free -SH groups into disulfide bonds [40]. On the contrary, UZMP–MD60 showed significantly higher free -SH groups compared to UZMP–MD45 (*p* < 0.05). This increase may be due to excessive ultrasound treatment, which promoted protein reaggregation and reduced the reactivity of free -SH groups toward oxidation or interaction with Maillard reaction products.

### 2.4. Physicochemical Properties of ZMP–MD Conjugates

#### 2.4.1. Sodium Dodecyl Sulfate–Polyacrylamide Gel Electrophoresis (SDS–PAGE)

**Figure 3:**
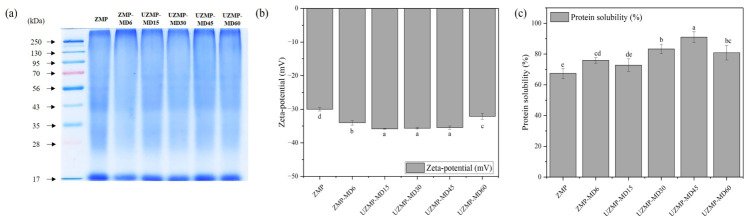
(**a**) SDS–PAGE, (**b**) zeta potential, and (**c**) protein solubility of ZMP, ZMP–MD6 conjugate, and UZMP–MD15, UZMP–MD30, UZMP–MD45, and UZMP–MD60 conjugates. Different lowercase letters indicate significant differences (*p* < 0.05) among all samples for each parameter.

SDS–PAGE analysis is an effective method for distinguishing changes in the molecular patterns of conjugates after Maillard glycosylation. As shown in Figure 3a, ZMP displayed characteristic molecular bands, containing between 28 and 35 kDa, 43 kDa, and 56 kDa. The obtained result is consistent with previous studies [6,8]. After conjugation with MD, either ZMP–MD or UZMP–MD conjugates showed different protein patterns compared to the native ZMP. For ZM–MD6, those major bands in ZMP were dramatically diminished, and the smear band showed stronger intensity at the top of the separating gel. Similarly, the UZMP–MDs conjugate groups also exhibited faint bands below 56 kDa and smear bands above 250 kDa under extended ultrasound treatment. These results confirmed that wet heating and ultrasound-assisted Maillard glycosylation produced a high molecular weight molecule via covalent bonding between ZMP and MD. In addition, the observed patterns align with previous findings by Chen et al. [19], who reported that ultrasound-assisted glycosylation of ovalbumin and dextran resulted in the appearance of high molecular weight bands in the gel.

#### 2.4.2. Zeta Potential

Zeta potential reflects the total surface charge of molecules, and a higher value indicates stronger electrostatic repulsion. As shown in Figure 3b, the absolute charge of the surface potential of ZMP–MD6 and UZMP–MD15–60 conjugates (−32.10 to −35.83 mV) was significantly higher than that of ZMP (−30.00 mV) (*p* < 0.05). No significant differences (*p* > 0.05) were observed among UZMP–MD15, UZMP–MD30, and UZMP–MD45, but UZMP–MD60 showed a significantly lower absolute value (−32.10 mV) compared to UZMP–MD45 (−35.83 mV) (*p* < 0.05). Here, maltodextrin (MD), a neutral polysaccharide, is considered to have no net charge [23]. Thus, the more negative zeta potential of the conjugates was unlikely to be primarily caused by MD itself. Instead, it may be associated with the Maillard glycosylation reaction between the -NH_2_ groups in ZMP and MD, reducing the cationic groups on ZMP’s surface [41]. Additionally, UZMP–MD15, 30, and 45 showed a more pronounced negative charge than ZMP–MD6, which could be explained by cavitation-induced bubble collapse during ultrasound treatment, protein unfolding, and exposure of more buried charged residues in ZMP [36]. However, UZMP–MD60 exhibited a reduced negative charge, which may be due to aggregation, as described in Section 2.3.3. Overall, appropriate ultrasound-assisted Maillard glycosylation can induce structural unfolding and potentially improve dispersion in aqueous solutions.

#### 2.4.3. Protein Solubility

The protein solubility of ZMP and ZMP–MD conjugates is shown in Figure 3c. The results showed that either wet heating (75.92%) or ultrasound-assisted Maillard glycosylation (72.79–90.99%) significantly increased protein solubility compared to ZMP (67.47%). This phenomenon could be attributed to the introduction of hydrophilic groups of MD into ZMP after conjugation. Moreover, UZMP–MD 30 and 45 exhibited better protein solubility than ZMP–MD6. The cavitation phenomenon during ultrasound treatment produced numerous collapsing bubbles that elevated local temperature and pressure, thereby inducing protein unfolding, disruption of peptide bonds, and exposure of inner hydrophilic amino acid residues, ultimately enhancing protein solubility [21]. Furthermore, the observed trends in protein solubility of UZMP–MD conjugate groups were in line with the hypothesis presented in the zeta potential analysis. The increased absolute zeta potential values correlated with the enhanced dispersion of UZMP-MD 15, 30, and 45. However, UZMP–MD60 exhibited decreased protein solubility, as evidenced by a reduced absolute zeta potential with increasing ultrasound treatment time. In summary, the formation of conjugates with MD under appropriate ultrasound-assisted conditions can improve the solubility of ZMP.

### 2.5. Acid-Induced Gel Properties of ZMP–MD Conjugates

**Figure 4:**
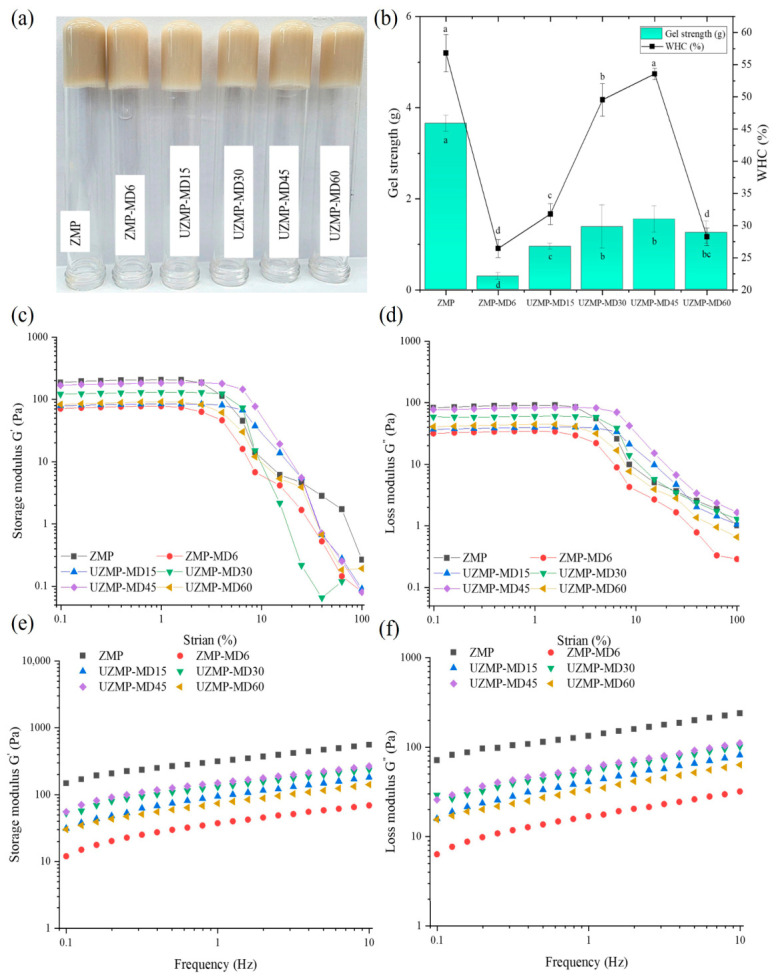
(**a**) The apparent morphology of acid-induced gels, (**b**) gel strength and water-holding capacity, (**c**,**d**) storage and loss modulus strain sweeps, (**e**,**f**) storage and loss modulus of frequency sweeps of ZMP, ZMP–MD6 conjugate, and UZMP–MD15, UZMP–MD30, UZMP–MD45, and UZMP–MD60 conjugates. Different lowercase letters indicate significant differences (*p* < 0.05) among all samples for each parameter.

In this study, the acid-induced gels were prepared using the conjugates obtained from wet heating and ultrasound-assisted treatment (Figure 4a). Several analyses, including gel strength, water-holding capacity (CHC), and rheological properties, were evaluated for ZMP–MD and UZMP–MD gels and compared with those of the ZMP gel.

#### 2.5.1. Gel Strength and Water-Holding Capacity of the Gels

The strength and WHC of ZMP and ZMP–MD conjugate gels are shown in Figure 4b. As a result, ZMP–MD6 gel had a significantly lower gel strength (0.29 g) and WHC (26.45%) compared to ZMP gel (3.65 g and 56.81%) (*p* < 0.05). These results suggest that glycosylation with MD under the wet heating system has a disadvantage on the acid-induced gelation properties of ZMP. As shown in Figure 2c,d, a significant reduction in surface hydrophobicity and free -SH groups in ZMP–MD6 could be attributed to the formation of a loose gel network structure. Meanwhile, UZMP-MD conjugate gels increased in gel strength and WHC from 0.94 g and 31.81% to 1.54 g and 53.58%, after 45 min of ultrasound treatment, and then decreased to 1.25 g and 28.27%, respectively. Although the covalent grafting with MD under the ultrasound systems decreased the amount of available free -SH groups, cavitation effects induced structural transformation, leading to protein unfolding with increased hydrophobicity. These changes could contribute to increased molecular crosslinking in the UZMP-MD gels, forming a more compact gel structure than in ZMP–MD6. In addition, it should be noted that the WHC of UZMP-MD45 gel (53.58%) was comparable to that of the ZMP gel (56.81%) (*p* > 0.05). This phenomenon indicates that the new inverted hydroxyl groups of MD in the conjugate enabled the binding of more water within the gel system, leading to an increase in WHC [42]. However, the excessive ultrasound treatment (60 min) might have caused protein structure to refold, reducing hydrophobic interactions and thereby decreasing gel strength and WHC.

#### 2.5.2. Rheological Properties of Gels

The rheological properties of ZMP, ZMP–MD, and UZMP–MD gels are shown in Figure 4c–f. The dynamic strain sweep experiments were employed on the gel samples to determine the linear viscoelastic region (LVR) under the applied strain amplitudes. As shown in Figure 4c,d, the G′ and G″ of all the gels exhibited similar behavior, with a linear viscoelastic trend at low strains (0.1–1%). Based on the observed results, a 0.1% strain was selected for the subsequent frequency-sweep tests (Figure 4e,f). A frequency sweep was performed within the range of 0.1–10 Hz, which showed that the storage modulus (G′) was higher than the loss modulus (G″) in all gels, suggesting solid-like characteristics [43]. In addition, the slight frequency dependence observed in all gels suggests that ZMP and ZM–MD conjugate gels behave as physical gels, primarily relying on non-covalent interactions [44]. From that point of view, the G′ and G″ of ZMP–MD6 were significantly lower than those of the ZMP gels, implying that the wet heating Maillard glycosylation weakened the gel structure of ZMP. The main reason could be reduced surface hydrophobicity in the conjugate, along with increased hydrophilicity and steric hindrance from the grafted MD [15]. These changes inhibit hydrophobic interactions between ZMP, which are instrumental in the formation of a dense and cohesive gel network [45]. In addition, the decrease in free -SH groups may also inhibit further crosslinking between the protein via the sulphydryl–disulfide (SH/S–S) interchange during gelation, leading to weakening of the gel [46]. In contrast, ultrasound treatment for 15–45 min significantly increased the G′ and G″ of the UZMP–MD gels. The cavitation effect can facilitate ZMP unfolding, leading to a further increase in the number of hydrophobic residues on the surface of ZMP, and strengthening the gel network of UZMP-MD conjugates. Meanwhile, the decrease in G′ and G″ for UZMP–MD60 may be related to the embedding state of the hydrophobic groups following excessive ultrasound treatment. This was confirmed by the results of CD, surface hydrophobicity, zeta potential, and protein solubility. Thus, although conjugation with MD weakens the gel properties compared to native ZMP, an appropriate ultrasound-assisted Maillard glycosylation time can effectively enhance the gelation behavior of the conjugates.

## 3. Conclusions

In the present study, it was confirmed that a proper ultrasound-assisted Maillard glycosylation time accelerated DG and reduced the browning intensity of the UZMP–MD conjugates compared to the conjugate obtained via wet heating. In addition, the ultrasound treatment time significantly influenced the structural and physicochemical properties of UZMP–MD conjugates. For the acid-induced gel application of either ZMP–MD or UZMP–MD conjugates, the conjugation with MD resulted in a weakening of the gel network compared to the native ZMP. Nevertheless, ultrasound treatment (100 kHz, 450 W, 70 °C) for 45 min promoted protein unfolding, leading to increased surface hydrophobicity and enhanced gel strength, WHC, and rheological properties. Thus, appropriate ultrasound-assisted Maillard glycosylation can efficiently form conjugates, alter the structure of ZMP–MD conjugates, and contribute to regulating gel properties. Therefore, further investigation into optimal conditions for producing UMZP–MD conjugates is necessary to improve gel properties beyond those of protein-based gels.

## 4. Materials and Methods

### 4.1. Materials and Reagents

The dried *Zophobas morio* was bought from the Gochang Bio-insect farm (Gochang-gun, Jeollabuk-do, Republic of Korea). The sample was pulverized using a grinder (RT-04, Mill powder, Tainan, Taiwan) for 30 s and sieved (850 μm aperture), placed in an air-tight bag, and kept at −18 °C until further use. Maltodextrin (MD) with a dextrose equivalent (DE) range of 14.0–19.9 was purchased from ES Foods (Gunpo, Republic of Korea). Other applied chemicals in this study were of analytical grade and were ordered from Duksan Chemicals (Ansan, Republic of Korea)

### 4.2. Preparation of ZMP–MD Conjugates

#### 4.2.1. Preparation of Zophobas Morio Protein (ZMP)

The ground sample was mixed with ethanol at a ratio of 1:10 (*w*/*v*) to remove lipids at 40 °C in a shaking incubator (SI-600R, Jeio Tech, Daejeon, Republic of Korea) at 200 rpm for 1 h. The supernatant was decanted from the mixture using filter cloth, and the residue was dried in a fume hood under ambient conditions. This process was carried out twice, and the defatted sample was stored at −18 °C until protein extraction.

ZMP was prepared using alkaline extraction and acidic precipitation methods outlined in the previous study [6]. Briefly, the defatted powder was extracted with 0.25 M NaOH (1:100, *w*/*v*) in a water bath (GO-90 W, Jeio Tech) at 45 °C for 2.5 h. After extraction, the supernatant was collected by centrifugation (Laborgene 1248R, Labogene Co., Ltd., Daejeon, Republic of Korea) at 6000 rpm for 10 min at 4 °C. Subsequently, the ZMP was precipitated by adjusting the pH of the obtained supernatant to 4.4 using 1 M HCl, and then centrifuged at 6000 rpm for 10 min. The collected precipitate was further dissolved in distilled water and neutralized to pH 7 with 1 M NaOH. The protein extract was frozen overnight (−80 °C), freeze-dried (FDS8518, Ilsin BioBase Co., Ltd., Dongducheon-si, Republic of Korea), and the obtained ZMP powder was maintained at −18 °C until further experiments. The extraction yield of ZMP was calculated as 35.29 ± 0.90% based on the defatted powder weight.

#### 4.2.2. Ultrasound-Assisted Maillard Glycosylation

The ultrasound-assisted conditions, such as ultrasound power (450 W) and temperature (70 °C), reported by Ma et al. [20] were selected for Maillard glycosylation in this study. Preliminary experiments confirmed that these conditions were also suitable for ZMP–MD conjugation without excessive protein denaturation. Briefly, ZMP (1%, *w*/*v*) was prepared by stirring in distilled water with a magnetic bar at 25 °C for 2 h. Next, MD was added to the ZMP solution at a 1:1 (*w*/*w*) ratio and stirred at 25 °C for 2 h. After mixing, the pH of the mixture was adjusted to 9.0 with 1 M NaOH. Then, the mixture was transferred to the ultrasound bath equipped with a circulating cooling device (Kyung Il Ultrasonic, KHC-1SUMP, Ansan, Republic of Korea). The ultrasound-assisted glycosylation reaction was performed for 15, 30, 45, and 60 min, respectively. Immediately, the reaction mixture was cooled in an ice bath, then dialyzed using a 3.5 kDa molecular weight cut-off membrane. The dialysis was conducted at 4 °C for 24 h, with continuous stirring, and the distilled water was changed four times per day. The resulting solution was freeze-dried, and the obtained conjugate samples were denoted as UZMP–MD15, UZMP–MD30, UZMP–MD45, and UZMP–MD60, respectively.

#### 4.2.3. Conventional Wet Heating Maillard Glycosylation

The conventional glycosylation method was employed, as described in our previous methods, with modifications [47]. The preparation of the sample mixture containing ZMP and MD before reaction under heating is the same as described in Section 4.2.2. The adjusted pH of the mixture was placed in a water bath and heated at 70 °C for 6 h. After the reaction, the mixture was cooled, dialyzed, and freeze-dried as previously described. Then, the obtained conjugate by the conventional method was referred to as ZMP–MD6 throughout this study.

### 4.3. Degree of Grafting (DG)

The DG of conjugates obtained from different Maillard glycosylation methods was determined by using the o-Phthaldialdehyde (OPA) method, as described by Chen et al. [19]. The DG was calculated as follows:

(1) $$\mathrm{DG}\;(\%)\;=\;\frac{A_{0}-A_{1}}{A_{0}}\times 100$$

where *A*_0_ and *A*_1_ indicate the absorbance value of ZMP and conjugates, respectively.

### 4.4. Determination of the Maillard Glycosylation Process

The measurement of intermediate and advanced Maillard reaction products was performed according to the method of Li et al. [48] with slight modifications. The obtained conjugate samples were dissolved in distilled water and appropriately diluted to a protein concentration of 2 mg/mL. Then, the absorbance of the solutions was recorded at 294 nm (A_294_) and 420 nm (A_420_) using a UV/Vis spectrophotometer (UV-2075 Plus, JASCO International Co., Ltd., Tokyo, Japan), respectively.

### 4.5. Structural Analysis

#### 4.5.1. FT-IR

All conjugates and ZMP samples (2 mg) were used in FT-IR analysis using an FTIR spectrophotometer (Frontier, PerkinElmer, Waltham, MA, USA). The spectra of FT-IR were scanned from 4000 cm^−1^ to 400 cm^−1^ with 16 scans at a resolution of 4 cm^−1^.

#### 4.5.2. Secondary Structure

The secondary structure of ZMP and ZMP–MD conjugates was determined using the method reported by Ma et al. [20]. Briefly, a protein concentration of 0.25 mg/mL in 10 mM phosphate-buffered saline (PBS) (pH 7.0) was prepared and transferred to a square-quartz cuvette with a Teflon cap of 5 mm path length. The CD spectra of the samples were measured in the far-UV region from 190 to 250 nm using a J-1500 spectropolarimeter (JASCO, Japan) with 5 scans at 50 mm/min, a bandwidth of 0.5 nm. Secondary structure contents were calculated using the Dichroweb server (https://bio.tools/dichroweb, accessed on 17 December 2025) with the CONTIN algorithm and the reference dataset SP175 (optimized for soluble proteins in the 190–240 nm range) [7].

#### 4.5.3. Surface Hydrophobicity

The surface hydrophobicity of ZMP, ZMP–MD, and UZMP-MDs was determined using the bound bromophenol blue (BPB) method as described in the previous method [6]. The samples were dissolved in 10 mM PBS (pH 7.0) at a protein concentration of 1 mg/mL. Then, 200 μL of BPB solution (1 mg/mL in distilled water) was added to the sample solutions and reacted at 25 °C for 10 min. Subsequently, the mixtures were centrifuged at 2000 rpm for 20 min to collect the supernatants. After a 10-fold dilution with distilled water, the absorbance of the supernatants was measured at 595 nm using a SPECTROstar Nanomicroplate reader (BMG LABTECH, Ortenberg, Germany). The control was prepared using distilled water instead of the sample. The amount of BPB (μg) attached to the sample’s surface reflects surface hydrophobicity and was calculated as follows:

(2) $$\mathrm{Bound}\;\mathrm{BPB}\;(\mu g)\;=\;\frac{{(A}_{0}-A_{1})\times 200}{A_{0}}$$

where *A*_0_ and *A*_1_ are the absorbance values of the control and sample, respectively.

#### 4.5.4. Free Sulfhydryl Group (-SH)

The free -SH groups content of the ZMP and ZMP–MD conjugates was determined using Ellman’s reagent (DTNB) following the method of Fu et al. [49]. Briefly, the samples were dissolved in Tris-glycine buffer (pH 8.0), containing 86 mM Tris, 90 mM glycine, and 3 mM EDTA, to a final concentration of 2 mg/mL. The mixtures were incubated in a shaking incubator at 25 °C for 2 h, followed by centrifugation at 10,000 rpm at 4 °C for 20 min. Then, the 3 mL of the recovered supernatants were further mixed with 30 μL of Ellman’s reagent and reacted in a dark room at 25 °C for 20 min. The absorbance of the mixtures was measured using a UV spectrophotometer at 420 nm. The buffer without a sample was used as a blank. The free -SH content was calculated as follows:

(3) $$\mathrm{SH}\;(\mu \mathrm{mol}/g)\;=\;\frac{{73.53\times D\times A}_{413}}{C}$$

where *A*_412_ is the absorbance value of the samples at 412 nm, *D* is the dilution factor, *C* is the sample concentration (mg/mL), and the factor 73.53 is obtained from 10^6^/(1.36 × 10^4^); 1.36 × 10^4^ = the molar absorptivity constant (M^−1^ cm^−1^) [50].

### 4.6. Physicochemical Analysis

#### 4.6.1. SDS-PAGE

SDS–PAGE was performed in accordance with the method described by Chailangka et al. [16] with modifications. ZMP and ZMP–MD conjugates were dissolved in RO–PREPTM 155 Protein Extraction Solution (iNtRON Biotechnology, Seoul, Republic of Korea) to a final concentration of 2 mg/mL. Then, the sample solutions were mixed with SDS buffer (Biosesang, Yongin, Republic of Korea) at a 4:1 ratio, and the mixtures were subsequently heated at 100 °C for 10 min. Next, 20 μL of sample mixtures were loaded into the well, consisting of a 5% stacking gel and a 10% separating gel. The electrophoresis was carried out at 90 V for 2 h. The obtained gel was stained with Coomassie Brilliant Blue overnight and de-stained using a solution of ethanol, distilled water, and acetic acid (4.5:4.5:1, *v*/*v*/*v*). The protein molecular weight bands were compared to the Xpert Prestained Protein Marker (GenDEPOT, Barker, TX, USA).

#### 4.6.2. Zeta Potential

The zeta potential values of ZMP, ZMP–MD, and UZMP-MDs were determined using a Zetasizer Nano ZS instrument (Malvern Panalytical, Malvern, UK) following the method of Cho et al. [6]. All the samples were prepared by dissolving them in 10 mM PBS (pH 7.0) at a sample concentration of 1 mg/mL before measurement.

#### 4.6.3. Protein Solubility

The PS was analyzed according to the reported method by Li et al. [21] with slight modifications. All samples were dispersed in distilled water and diluted to obtain a protein concentration of 2 mg/mL. The sample solutions were centrifuged at 8000 rpm for 15 min at 4 °C. Afterward, the protein content in the supernatant was measured at 595 nm using the Bradford protein assay with a UV spectrophotometer, with bovine serum albumin (BSA) used as a standard. The PS of the sample was calculated as follows:

(4) $$\mathrm{Protein}\;\mathrm{solubility}\;(\%)\;=\;\frac{P_{1}}{P_{0}}\times 100$$

where *P*_1_ and *P*_0_ represent the protein content in the sample (mg/mL) and the initial protein concentration, respectively.

### 4.7. Gel Properties

#### 4.7.1. Preparation of Acid-Induced Gels

The acid-induced gels of ZMP, ZMP–MD, and UZM–MDs were prepared according to the method reported by Zhao et al. [14] with slight modifications. Either ZMP or conjugate samples were dissolved in distilled water at 8% (*w*/*v*) and stirred magnetically at 25 °C for 2 h. After stirring, GDL was added to the protein or conjugate dispersion at a mass ratio of 10:1, and the mixture was stirred for an additional 5 min to ensure homogeneity. The resulting mixtures were maintained at 25 °C for 2 h to acidify and kept overnight at 4 °C to form a gel.

#### 4.7.2. Gel Strength

The gel strength of the acid-induced gels was measured using a texture analyzer (Compac-100D rheometer, Sun Scientific Co., Tokyo, Japan) equipped with a 2 kg load cell. The gels with a diameter of 35 mm and a height of 10 mm were compressed to a depth of 30% at a table speed of 60 mm/min using a cylindrical probe (20 mm diameter). The gel strength (g) corresponded to the maximum force required to penetrate the gel.

#### 4.7.3. Water-Holding Capacity (WHC)

The WHC of the acid-induced gels was determined using the centrifugation method reported by Said et al. [47]. The gel samples (5 g) were transferred into a 50 mL centrifugal tube and centrifuged at 4000 rpm for 20 min at 4 °C. After centrifugation, the released water was carefully removed. The WHC was calculated following the equation:

(5) $$\mathrm{WHC}\;(\%)\;=\;\frac{W_{3}-W_{2}}{W_{1}}\times 100$$

where *W*_1_ is the initial gel weight (g), *W*_2_ is the empty tube weight (g), and *W*_3_ is the tube + gel after centrifugation (g).

#### 4.7.4. Rheological Properties

The rheological behavior of the produced acid-induced gels was investigated using a Haake Mars Modular Advanced Rheometer System (Thermo Scientific Haake Mars III Controller, Waltham, MA, USA) fitted with a parallel-plate geometry (P35 Ti L, 1 mm gap) at 25 °C. The linear viscoelastic region (LVR) was first identified over a strain range of 0.1–100% at a frequency of 1.0 Hz. Subsequently, the frequency-sweep test was performed, ranging from 0.1 to 10 Hz. At that same time, the storage modulus (G′) and loss modulus (G″) were recorded [51].

### 4.8. Statistical Analysis

All experiments were conducted in triplicate, and results are reported as mean ± standard deviation (SD). Means were compared using Duncan’s multiple range test in SPSS v.22 (SPSS Inc., Chicago, IL, USA) at a significance level of 5% (*p* < 0.05).

## Figures and Tables

**Table 1:** The secondary structure compositions of ZMP, ZMP–MD6 conjugate, and UZMP–MD15, UZMP–MD30, UZMP–MD45, and UZMP–MD60 conjugates.

Sample	α-Helix (%)	β-Sheet (%)	β-Turn (%)	Random Coil (%)
ZMP	26.97 ± 0.80 a	21.67 ± 1.76 d	12.80 ± 0.53 ab	38.63 ± 0.64 cd
ZMP–MD6	23.37 ± 2.40 b	22.40 ± 0.80 d	13.50 ± 0.42 a	40.70 ± 2.87 bc
UZMP–MD15	23.35 ± 0.25 b	27.20 ± 1.40 c	12.10 ± 0.00 b	37.30 ± 1.20 d
UZMP–MD30	4.20 ± 0.10 d	44.00 ± 0.00 a	9.50 ± 0.00 d	42.40 ± 0.10 b
UZMP–MD45	4.10 ± 1.10 d	39.60 ± 3.50 b	10.55 ± 1.25 c	45.80 ± 1.20 a
UZMP–MD60	20.80 ± 0.50 c	27.05 ± 0.35 c	12.00 ± 0.10 b	40.15 ± 0.05 bc

Values represent mean ± SD (*n* = 3). Different letters in a column indicate significantly different (*p* < 0.05).

## Data Availability

The original contributions presented in this study are included in the article. Further inquiries can be directed to the corresponding authors.

## Abbreviations

The following abbreviations are used in this manuscript.

ZMP	*Zophobas morio* protein
MD	Maltodextrin
ZMP–MD	*Zophobas morio* protein–maltodextrin conjugate
UZMP-MD	Ultrasound-assisted ZMP–MD conjugate
GDL	Glucono-δ-lactone
DG	Degree of grafting
MR	Maillard reaction
FT-IR	Fourier transform infrared spectroscopy
CD	Circular dichroism
SDS-PAGE	Sodium dodecyl sulfate–polyacrylamide gel electrophoresis
WHC	Water-holding capacity
SH	Sulfhydryl group
BPB	Bromophenol blue

## References

[1] Andrade R.C., de Carvalho Alves J., Roselino M.N. (2021). A review of *Zophobas morio*: Chemical, nutritional and functional characteristics. Adv. Food Sci. Technol..

[2] Čaloudová J., Křištofová K., Pospiech M., Klempová T., Slaný O., Čertík M., Marcinčák S., Makiš A., Javůrková Z., Pečová M. (2023). Effects of biofermented feed on *Zophobas morio*: Growth ability, fatty acid profile, and bioactive properties. Sustainability.

[3] Rumbos C., Athanassiou C. (2021). The superworm, *Zophobas morio* (Coleoptera: Tenebrionidae): A ‘sleeping giant’ in nutrient sources. J. Insect Sci..

[4] Zhatkanbayeva Z., Kurtibay K., Zhatkanbayev Y., Kappassuly A., Fedeli R. (2025). Chemical aspects of biomolecule extraction from *Zophobas morio* larvae: Lipid and protein extraction mechanisms and solvent efficiency analysis. Chem. J. Kazakhstan.

[5] Zielińska E. (2022). Evaluating the functional characteristics of certain insect flours (non-defatted/defatted flour) and their protein preparations. Molecules.

[6] Cho H.-S., Park J.-H., Olawuyi I.F., Nam J.-O., Lee W.-Y. (2025). Physicochemical characteristics and anti-inflammatory properties of *Zophobas morio* (super mealworm) protein extracted by different methods. Food Chem..

[7] Cho H.-S., Olawuyi I.F., Lee W.-Y. (2025). Comparative extraction and recovery of *Zophobas morio* protein using deep eutectic solvents and conventional alkaline methods: Effects on structure, functionality, and antioxidant properties. Int. J. Biol. Macromol..

[8] Cho H.-S., Park J.-H., Olawuyi I.F., Nam J.-O., Lee W.-Y. (2025). Optimization of ultrasound-assisted enzymatic hydrolysis *Zophobas morio* protein and its protective effects against H_2_O_2_-induced oxidative stress in RAW264. 7 cells. Int. J. Biol. Macromol..

[9] Scholliers J., Steen L., Fraeye I. (2020). Partial replacement of meat by superworm (*Zophobas morio* larvae) in cooked sausages: Effect of heating temperature and insect: Meat ratio on structure and physical stability. Innov. Food Sci. Emerg. Technol..

[10] Scholliers J., Steen L., Glorieux S., Van de Walle D., Dewettinck K., Fraeye I. (2019). The effect of temperature on structure formation in three insect batters. Food Res. Int..

[11] Cho H.-S., Olawuyi I.F., Park J.-J., Lee W.-Y. (2023). Quality characteristics of eggless muffins prepared using egg solution alternatives containing super mealworm protein isolate and carrageenan. Int. J. Food Sci. Technol..

[12] Pan J., Xu H., Cheng Y., Mintah B.K., Dabbour M., Yang F., Chen W., Zhang Z., Dai C., He R. (2022). Recent insight on edible insect protein: Extraction, functional properties, allergenicity, bioactivity, and applications. Foods.

[13] Sadeghi M., Madadlou A., Khosrowshahi A., Mohammadifar M. (2014). Acid-induced gelation behavior of casein/whey protein solutions assessed by oscillatory rheology. J. Food Sci. Technol..

[14] Zhao C., Yin H., Yan J., Niu X., Qi B., Liu J. (2021). Structure and acid-induced gelation properties of soy protein isolate–maltodextrin glycation conjugates with ultrasonic pretreatment. Food Hydrocoll..

[15] Spotti M.J., Loyeau P.A., Marangón A., Noir H., Rubiolo A.C., Carrara C.R. (2019). Influence of Maillard reaction extent on acid induced gels of whey proteins and dextrans. Food Hydrocoll..

[16] Chailangka A., Seesuriyachan P., Wangtueai S., Ruksiriwanich W., Jantanasakulwong K., Rachtanapun P., Sommano S.R., Leksawasdi N., Barba F.J., Phimolsiripol Y. (2022). Cricket protein conjugated with different degrees of polymerization saccharides by Maillard reaction as a novel functional ingredient. Food Chem..

[17] Mshayisa V.V., Van Wyk J. (2021). Hermetia illucens protein conjugated with glucose via maillard reaction: Antioxidant and techno-functional properties. Int. J. Food Sci..

[18] Chailangka A., Phongthai S., Leksawasdi N., Mousavi Khaneghah A., Bangar S.P., Phimolsiripol Y. (2024). Optimization of Ultrasound and Microbubble-Assisted Maillard Reaction on Conjugated Cricket Protein with Fructooligosaccharide. Food Bioprocess Technol..

[19] Chen B., Chen L., Li C., Huang W., Zhao Y., Ai C., Teng H. (2024). Ultrasound-assisted glycosylation of ovalbumin and dextran conjugate carrier for anthocyanins and their stability evaluation. Ultrason. Sonochem..

[20] Ma X., Hou F., Zhao H., Wang D., Chen W., Miao S., Liu D. (2020). Conjugation of soy protein isolate (SPI) with pectin by ultrasound treatment. Food Hydrocoll..

[21] Li C., Huang X., Peng Q., Shan Y., Xue F. (2014). Physicochemical properties of peanut protein isolate–glucomannan conjugates prepared by ultrasonic treatment. Ultrason. Sonochem..

[22] Loret C., Meunier V., Frith W.J., Fryer P.J. (2004). Rheological characterisation of the gelation behaviour of maltodextrin aqueous solutions. Carbohydr. Polym..

[23] Kim J.S., Do Kim H., Ye Y.J., Lee M.H. (2025). Enhanced emulsion stability through synergistic interactions in ternary Maillard conjugates of soy protein isolate, maltodextrin, and gum Arabic. Food Chem..

[24] Aziznia S., Askari G., Emamdjomeh Z., Salami M. (2024). Effect of ultrasonic assisted grafting on the structural and functional properties of mung bean protein isolate conjugated with maltodextrin through maillard reaction. Int. J. Biol. Macromol..

[25] Sun J., Mu Y., Mohammed O., Dong S., Xu B. (2020). Effects of single-mode microwave heating and dextran conjugation on the structure and functionality of ovalbumin–dextran conjugates. Food Res. Int..

[26] Li K., Wang J., Zhao P., McClements D.J., Liu X., Liu F. (2024). Effect of ultrasound-assisted Maillard reaction on glycosylation of goat whey protein: Structure and functional properties. Food Chem..

[27] He W., Tian L., Zhang S., Pan S. (2021). A novel method to prepare protein-polysaccharide conjugates with high grafting and low browning: Application in encapsulating curcumin. LWT.

[28] Chen X., Dai Y., Huang Z., Zhao L., Du J., Li W., Yu D. (2022). Effect of ultrasound on the glycosylation reaction of pea protein isolate–arabinose: Structure and emulsifying properties. Ultrason. Sonochem..

[29] Qu W., Zhang X., Chen W., Wang Z., He R., Ma H. (2018). Effects of ultrasonic and graft treatments on grafting degree, structure, functionality, and digestibility of rapeseed protein isolate-dextran conjugates. Ultrason. Sonochem..

[30] Chen W., Ma X., Wang W., Lv R., Guo M., Ding T., Ye X., Miao S., Liu D. (2019). Preparation of modified whey protein isolate with gum acacia by ultrasound maillard reaction. Food Hydrocoll..

[31] De Oliveira F.C., Coimbra J.S.d.R., De Oliveira E.B., Zuñiga A.D.G., Rojas E.E.G. (2016). Food protein-polysaccharide conjugates obtained via the Maillard reaction: A review. Crit. Rev. Food Sci. Nutr..

[32] Liu L., Li X., Du L., Zhang X., Yang W., Zhang H. (2019). Effect of ultrasound assisted heating on structure and antioxidant activity of whey protein peptide grafted with galactose. LWT.

[33] Xue F., Li C., Adhikari B. (2020). Physicochemical properties of soy protein isolates-cyanidin-3-galactoside conjugates produced using free radicals induced by ultrasound. Ultrason. Sonochem..

[34] Zhao C.-B., Zhou L.-Y., Liu J.-Y., Zhang Y., Chen Y., Wu F. (2016). Effect of ultrasonic pretreatment on physicochemical characteristics and rheological properties of soy protein/sugar Maillard reaction products. J. Food Sci. Technol..

[35] Qu W., Zhang X., Han X., Wang Z., He R., Ma H. (2018). Structure and functional characteristics of rapeseed protein isolate-dextran conjugates. Food Hydrocoll..

[36] Cui H., Zang Z., Jiang Q., Bao Y., Wu Y., Li J., Chen Y., Liu X., Yang S., Si X. (2023). Utilization of ultrasound and glycation to improve functional properties and encapsulated efficiency of proteins in anthocyanins. Food Chem..

[37] Han G., Li Y., Liu Q., Chen Q., Liu H., Kong B. (2022). Improved water solubility of myofibrillar proteins by ultrasound combined with glycation: A study of myosin molecular behavior. Ultrason. Sonochem..

[38] Chen L., Chen J., Wu K., Yu L. (2016). Improved low pH emulsification properties of glycated peanut protein isolate by ultrasound Maillard reaction. J. Agric. Food Chem..

[39] Nasrollahzadeh F., Varidi M., Koocheki A., Hadizadeh F. (2017). Effect of microwave and conventional heating on structural, functional and antioxidant properties of bovine serum albumin-maltodextrin conjugates through Maillard reaction. Food Res. Int..

[40] Xue H., Tu Y., Zhang G., Xin X., Hu H., Qiu W., Ruan D., Zhao Y. (2021). Mechanism of ultrasound and tea polyphenol assisted ultrasound modification of egg white protein gel. Ultrason. Sonochem..

[41] Zhao S., Huang Y., McClements D.J., Liu X., Wang P., Liu F. (2022). Improving pea protein functionality by combining high-pressure homogenization with an ultrasound-assisted Maillard reaction. Food Hydrocoll..

[42] Zhang Z., Arrighi V., Campbell L., Lonchamp J., Euston S.R. (2016). Properties of partially denatured whey protein products: Formation and characterisation of structure. Food Hydrocoll..

[43] Xi Z., Liu W., McClements D.J., Zou L. (2019). Rheological, structural, and microstructural properties of ethanol induced cold-set whey protein emulsion gels: Effect of oil content. Food Chem..

[44] Spotti M.J., Martinez M.J., Pilosof A.M., Candioti M., Rubiolo A.C., Carrara C.R. (2014). Influence of Maillard conjugation on structural characteristics and rheological properties of whey protein/dextran systems. Food Hydrocoll..

[45] Le Bon C., Nicolai T., Durand D. (1999). Growth and structure of aggregates of heat-denatured β-lactoglobulin. Int. J. Food Sci. Technol..

[46] Zhao C., Chu Z., Mao Y., Xu Y., Fei P., Zhang H., Xu X., Wu Y., Zheng M., Liu J. (2023). Structural characteristics and acid-induced emulsion gel properties of heated soy protein isolate–soy oligosaccharide glycation conjugates. Food Hydrocoll..

[47] Said N.S., Lee W.-Y. (2025). Development of soft food gels from high-methyl pectin-protein conjugates via acid-and heat-induced crosslinking for dysphagia-friendly applications. Int. J. Biol. Macromol..

[48] Li Z., Zheng Y., Sun Q., Wang J., Zheng B., Guo Z. (2021). Structural characteristics and emulsifying properties of myofibrillar protein-dextran conjugates induced by ultrasound Maillard reaction. Ultrason. Sonochem..

[49] Fu J.-j., Yu J.-x., He F.-y., Huang Y.-n., Wu Z.-p., Chen Y.-w. (2024). Physicochemical and functional characteristics of glycated collagen protein from giant salamander skin induced by ultrasound Maillard reaction. Int. J. Biol. Macromol..

[50] Ellman G.L. (1959). Tissue sulfhydryl groups. Arch. Biochem. Biophys..

[51] Hikmah S.N., Said N.S., Lee W.Y. (2025). Maillard-Type Conjugates of Pumpkin Seed Protein with Different Polysaccharides as Novel Emulsion Stabilizers: Structure–Function Relationships and Emulsifying Performance. Food Bioprocess Technol..

